# Using linked data to inform multidimensional real-world issues: Canadian examples

**DOI:** 10.23889/ijpds.v9i5.2715

**Published:** 2024-09-18

**Authors:** Winnie Chan, Li Xue

**Affiliations:** 1 Statistics Canada

Issues at the top of global and national policy agendas, such as the COVID-19 pandemic, climate emergency, and energy and cost of living crises, are illustrating the interconnectivity of the economy, society, and the environment. Moreover, it is clear, that impacts of these issues are not uniformly felt across societies. Greater granularity of information is required to address inequalities. As a result, policy makers are taking a more holistic view of issues to address the interlinkages across domains. This is driving the demand for Statistics Canada to provide statistical insights that address these cross-cutting policy issues and provide more granular information across multiple domains.

Linked data are necessary for understanding the interrelated nature of these issues and the scope of their impact. For example, recent Canadian research based on linked data has shown that COVID-19 led to changes in work arrangements that have implications for public transit use and greenhouse gas emissions. The employer-employee linked administrative data augmented with a further linkage to census data provide opportunities for Canadian researchers to produce multidimensional insights on a portrait of racialized groups and immigrants including refugees’ presence in the Canadian economy, their sociodemographic characteristics, and their performance over time relative to other Canadians, particularly post the COVID pandemic.

Rapid growth in data availability for research also poses new challenges ranging from technical issues needed for the feasibility of data linkage to stewardship issues related to governance, access, and oversight. The presentation will also discuss the lessons learned and reflections from the Canadian experiences.

